# Supplementary material for: Deterministic phase slips in mesoscopic superconducting rings
Source: Nat Commun. 2016 Nov 24;7:13551. doi: 10.1038/ncomms13551 (PMC5123074; doi:10.1038/ncomms13551)
Supplement: Supplementary Information — Supplementary Figures 1-9, Supplementary Table 1, Supplementary Notes 1-9 and Supplementary References [file ncomms13551-s1.pdf]

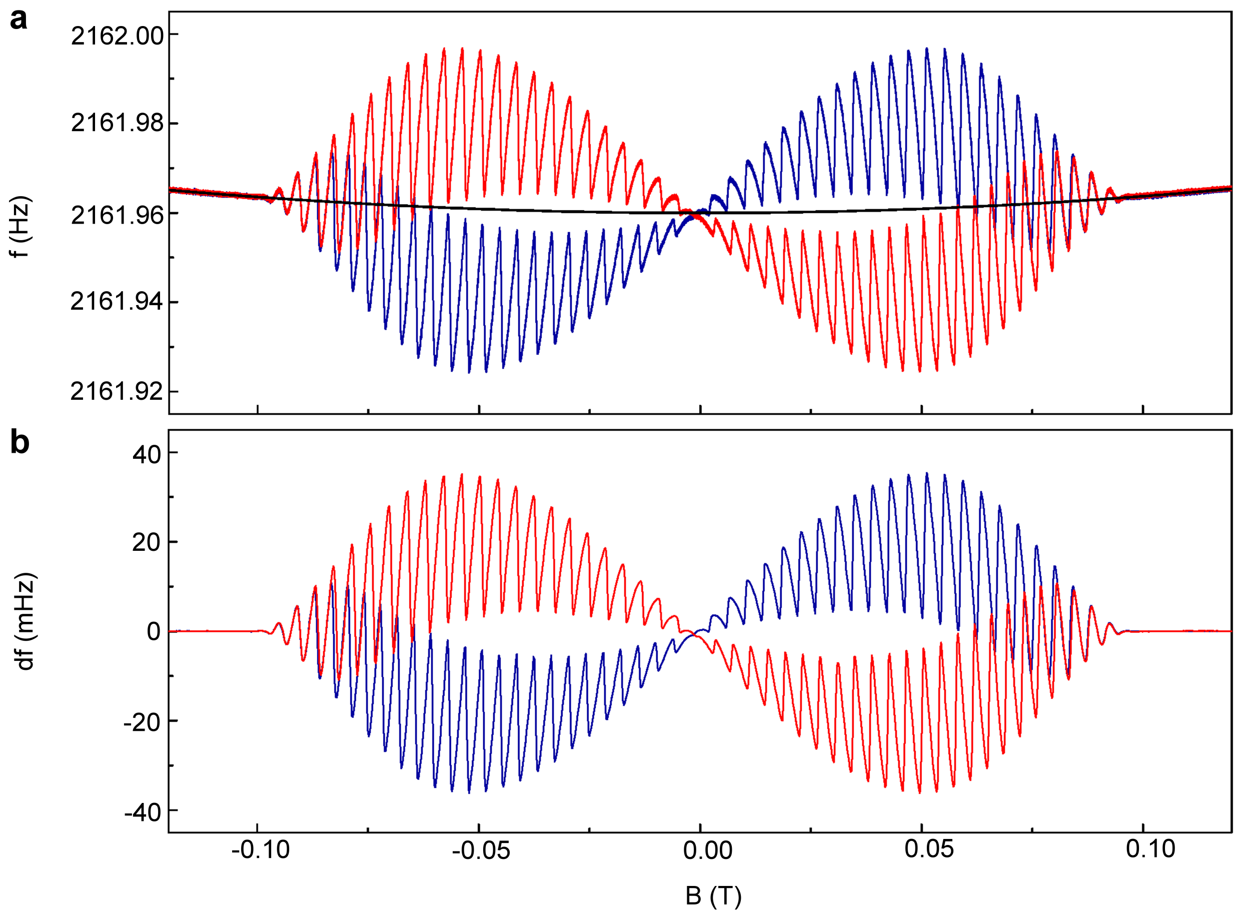

Supplementary Figure 1 | Background removal. (**a**) Raw data, cantilever frequency shift as function of field for increasing B (red) and decreasing B (blue) for the rings with radius R = 406 nm and T = 762 mK. Third order polynomial background is shown as the black curve. (**b**) Cantilever frequency shift after background substraction and averaging. The signal is due to the magnetic moment of the supercurrent.

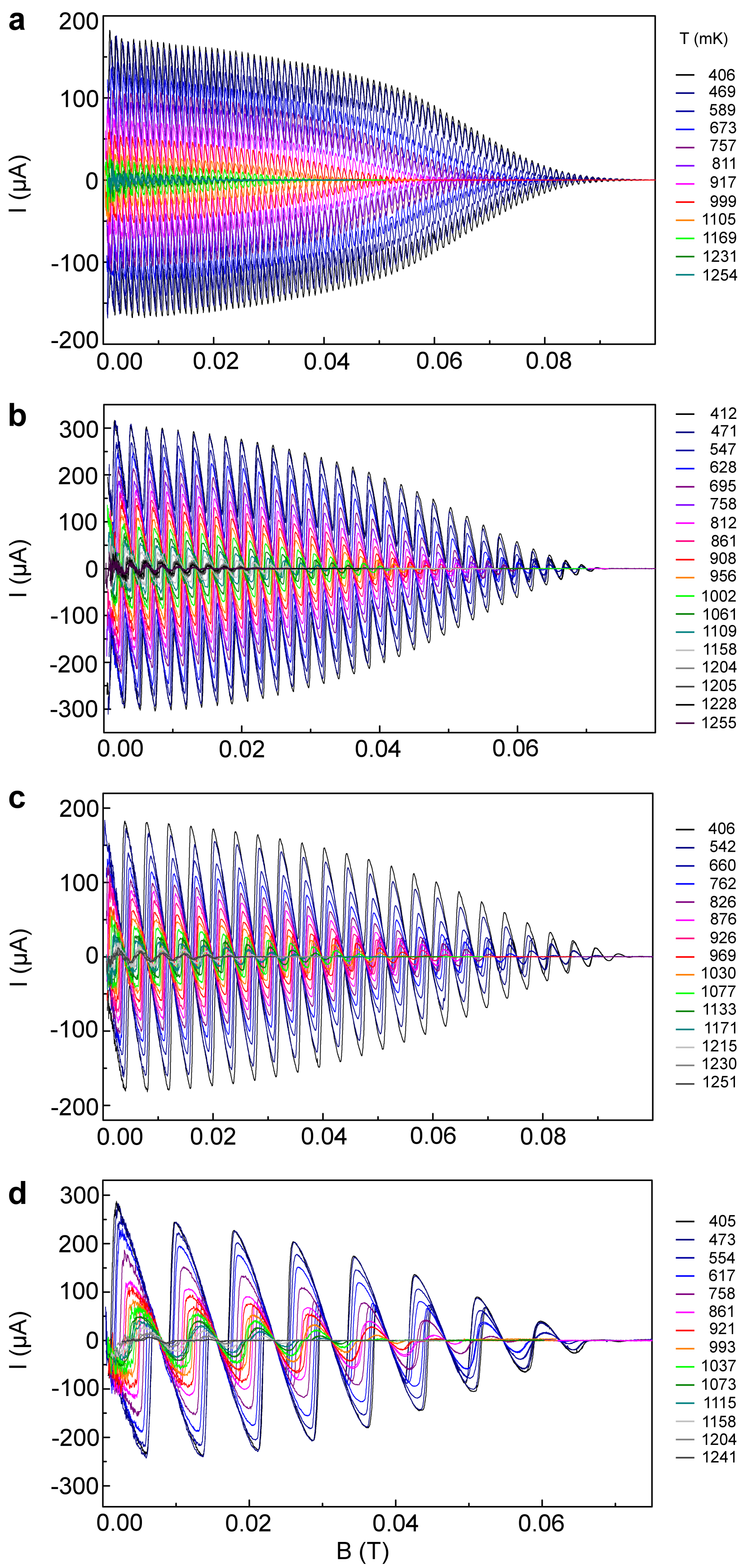

Supplementary Figure 2 | Measured supercurrent as function of field. Shown are different samples with $R = 780$, 538, 406, 288 nm ((**a**) to (**d**)), in the full temperature range. Lower curves of the same color correspond to increasing B and upper ones to decreasing B.

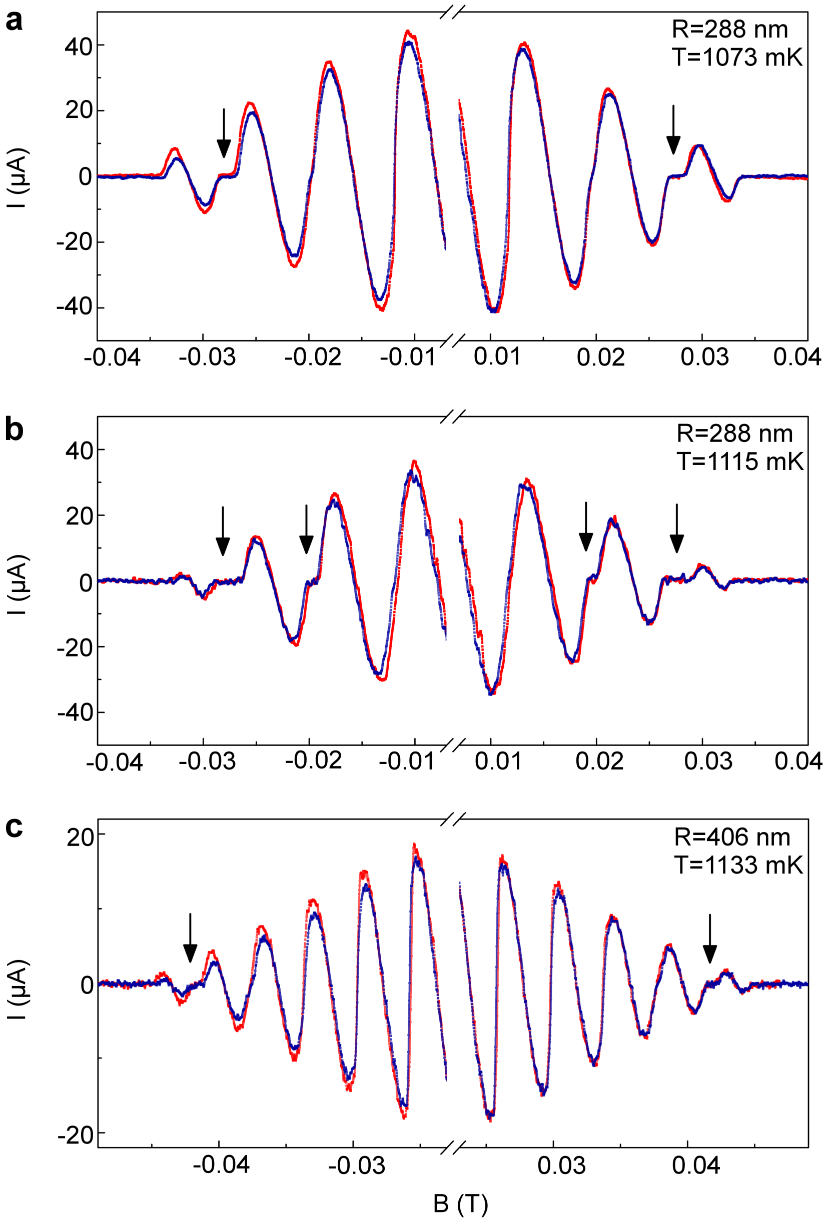

Supplementary Figure 3 | Little-Parks regime. Measured supercurrent $I(B)$ for ring sizes and temperatures marked in each panel. Normal state regions $(I = 0)$ are denoted by black arrows. Red curves correspond to sweep up and blue to sweep down.

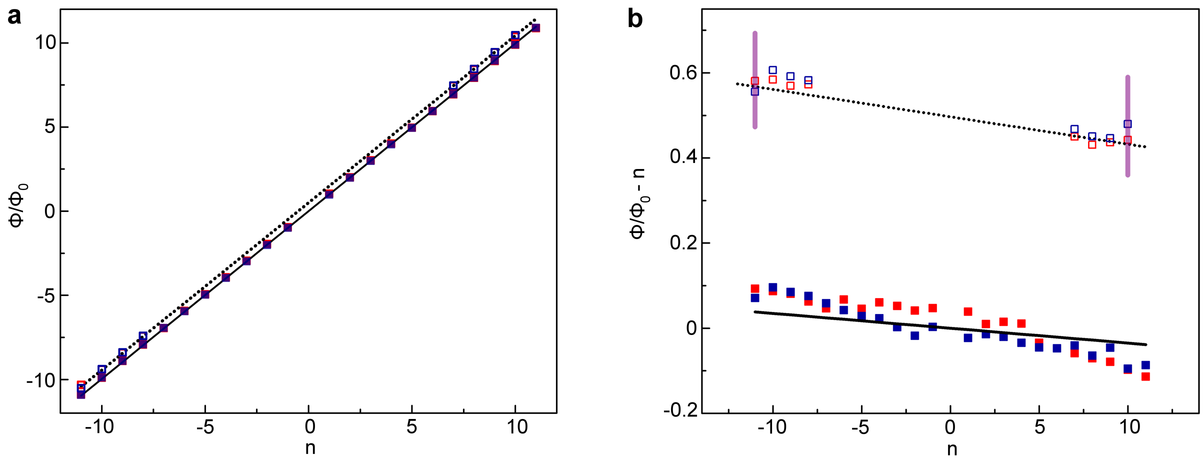

Supplementary Figure 4 | Quantization in the Little-Parks regime. Full squares denote normalized flux values at which current goes through zero, and empty squares denote normalized flux at which the winding number changes by one, both as function of winding number n. Black lines are the theoretical predictions for both cases, full and dotted respectively. In panel (**b**) with respect to (**a**) the linear background $\Phi/\Phi_0 = n$ is subtracted both from data and theoretical predictions, which enables to outline the trend due to finite-width correction. Purple vertical lines denote the normal-state regions. Red squares are for sweep up and blue for sweep down. Here $R = 406$ nm and $T = 1133$ mK.

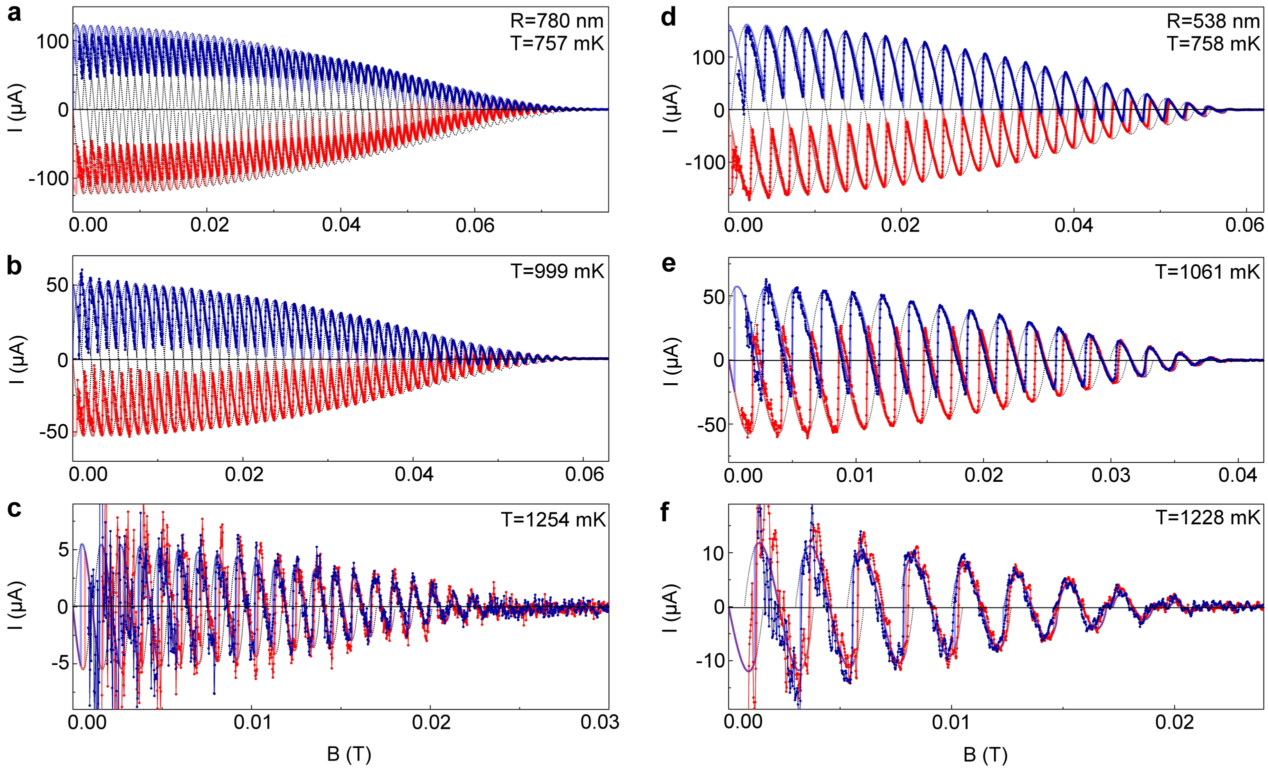

Supplementary Figure 5 | Supercurrent vs. field and Ginzburg-Landau fit. Data is shown for different ring sizes (columns) and temperatures (marked on each panel). Points and thin curves: data; thick curves: Ginzburg-Landau fit (see text). Red curves on each graph correspond to sweeping the field up, and the blue ones to sweeping down. Thin black dotted curves: the Ginzburg-Landau fit, extended over the full field range of each winding number.

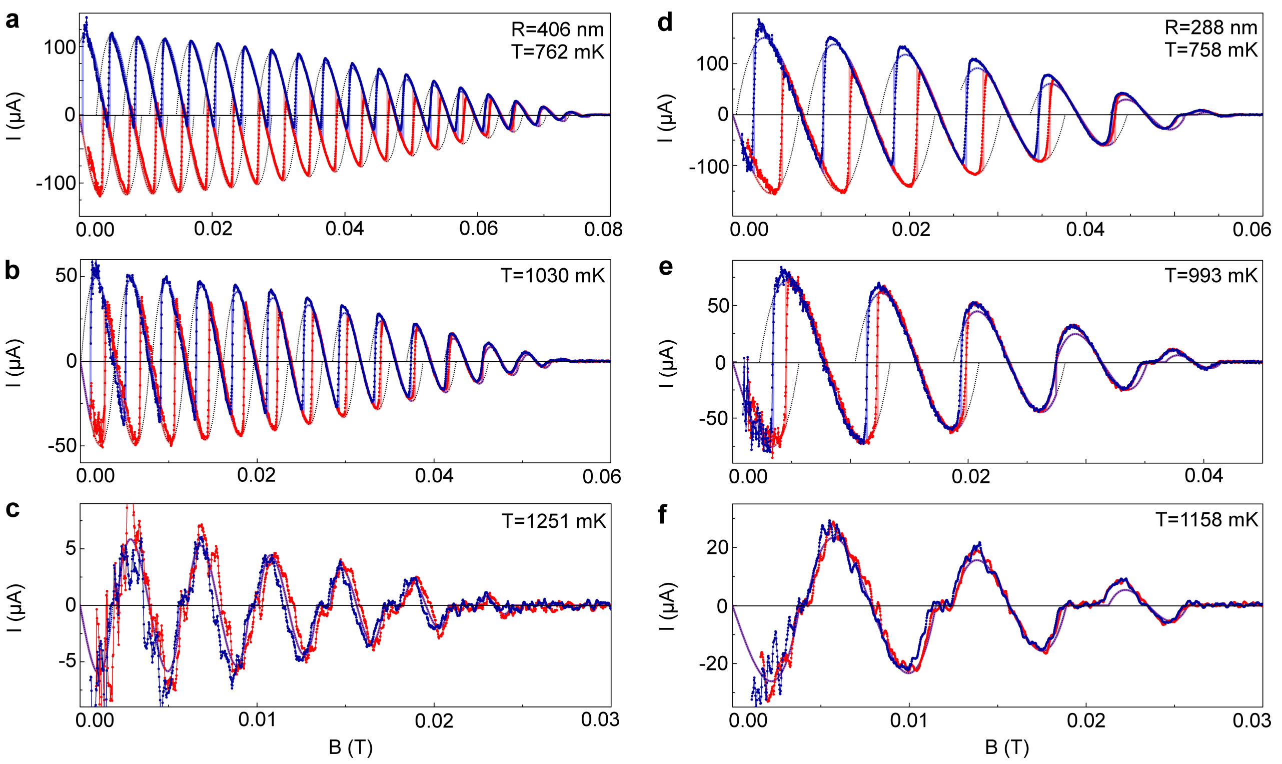

Supplementary Figure 6 | Supercurrent vs. field and Ginzburg-Landau fit. Data is shown for different ring sizes (columns) and temperatures (marked on each panel). Points and thin curves: data; thick curves: Ginzburg-Landau fit (see text). Red curves on each graph correspond to sweeping the field up, and the blue ones to sweeping down. Thin black dotted curves: the Ginzburg-Landau fit, extended over the full field range of each winding number.

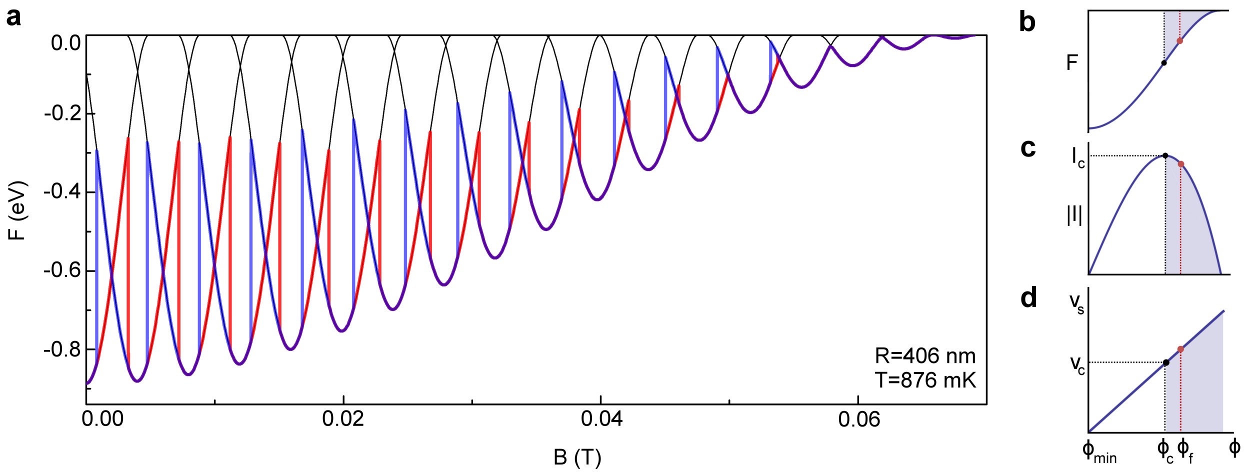

Supplementary Figure 7 | Free energy. (**a**) The black curve shows the equilibrium free energy F_n as a function of B for all n, for the ring with $R = 406$ nm at $T = 876$ mK. In this panel, the F_n are calculated from the fit parameters. The red (blue) curve shows the path followed by the system as B is increased (decreased). (**b**) Free energy of an equilibrium state as a function of flux. (**c**) Absolute value of supercurrent as a function of flux. (**d**) Velocity of the superconducting condensate as a function of flux. The black dots in panels **b**, **c** and **d** denote instability points ϕ_c, and red dots ϕ_f (see main text). Shaded regions are unstable in the sense specified in the text.

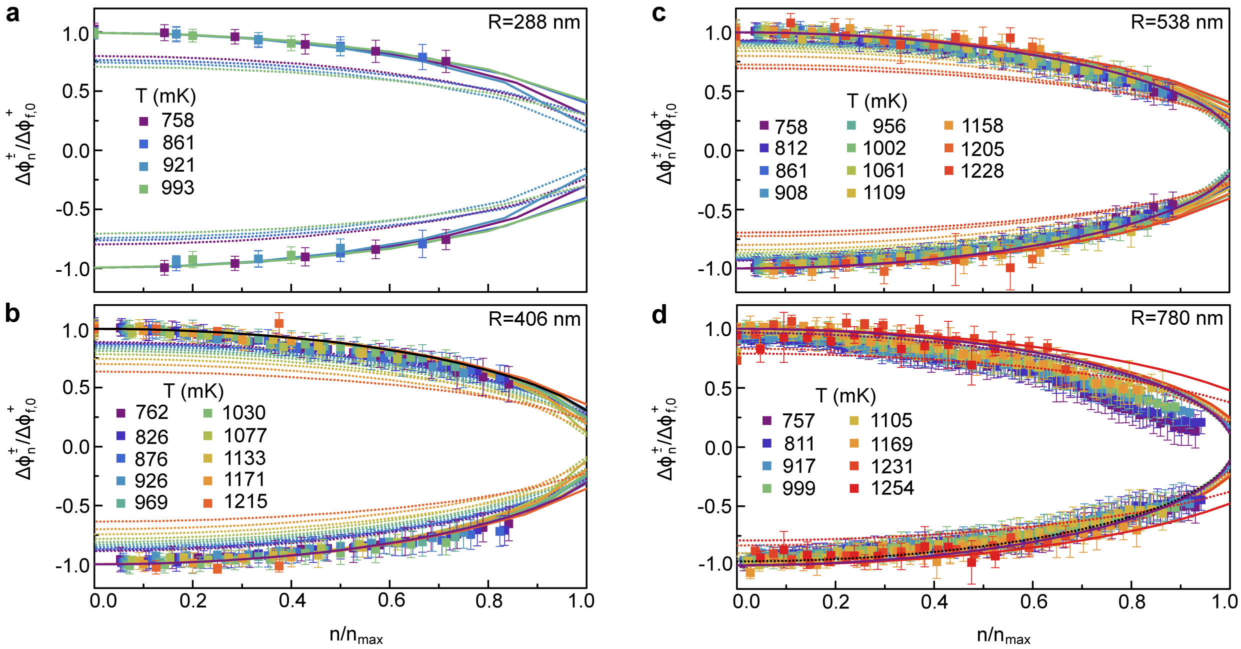

Supplementary Figure 8 | Phase slip flux as function of winding number. Dots: experimental values; bars: observed width of each jump; full lines: prediction for the switching flux $\Delta\phi^{\pm}_{\mathrm{f},n}$; dotted lines: prediction for the switching flux $\Delta\phi^{\pm}_{\mathrm{c},n}$ (see main text). Colors represent temperature. Radii R = 288, 406, 538, 780 nm of four different samples are denoted on the panels. The normalization of the axes is explained in the main text.

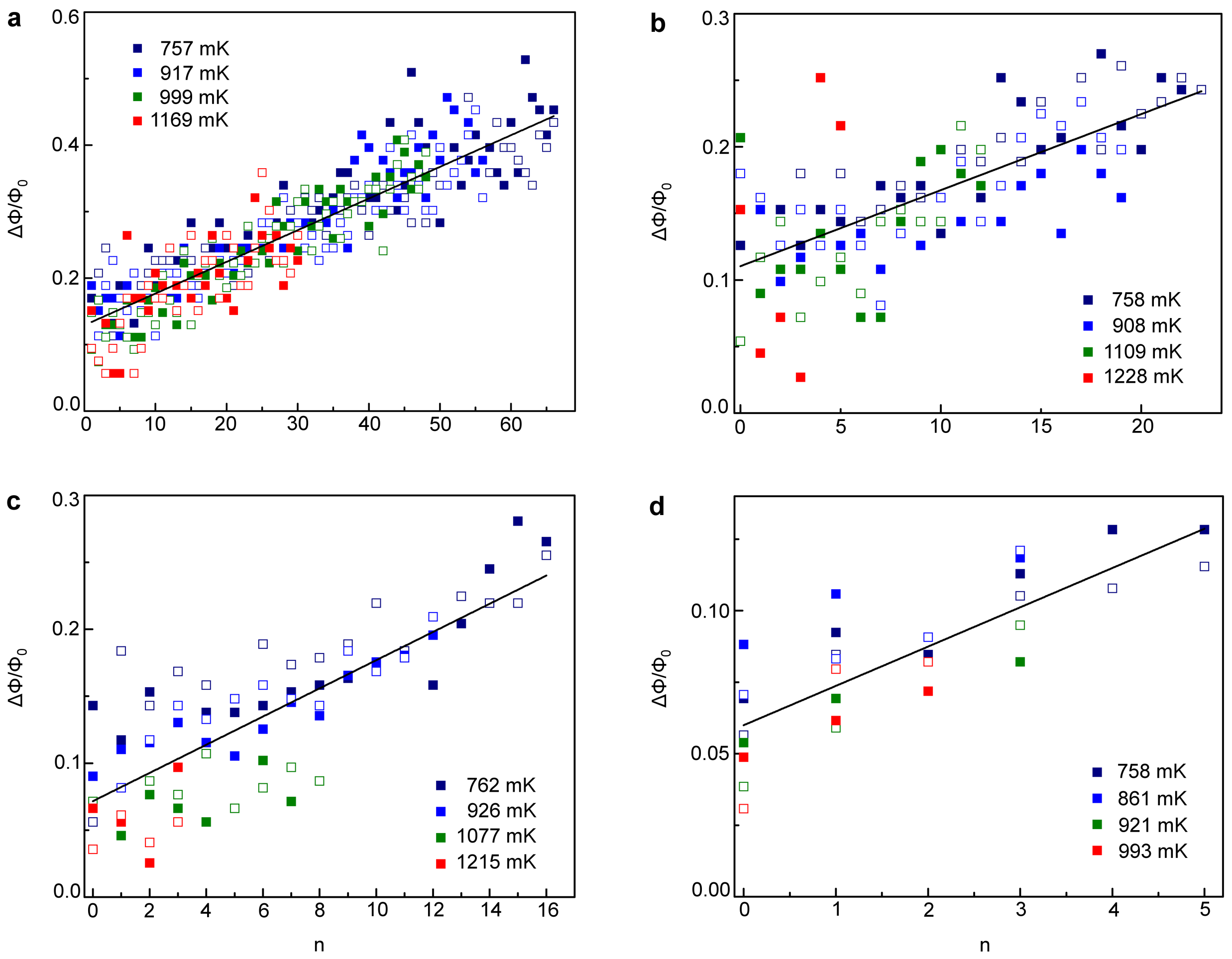

Supplementary Figure 9 | Transition width. Observed transition width in units of normalized flux $\Delta\Phi/\Phi_0$ as function of winding number n, for different ring sizes and temperatures. Solid lines indicate a linear fit to the whole data set at each ring size. Radius sizes are: (**a**) $R = 780$ nm, (**b**) $R = 538$ nm, (**c**) $R = 406$ nm and (**d**) $R = 288$ nm.

N^o	$R_{\rm nom}$ (nm)	$R_{\rm GL}$ (nm)	$w_{\rm nom}$ (nm)	$w_{\rm GL}$ (nm)	N	ξ_0 (nm)	λ_0 (nm)	$\lambda_{\rm P0}$ (nm)	$B_{\rm c3,0}$ (T)	$B_{\rm c3,0}^{\rm GL}$ (T)
1	250	288	80	65	1680	214(2)	97(1)	104(2)	0.0796(6)	0.087(1)
2	375	406	65	48	990	202(2)	95(1)	100(2)	0.1107(7)	0.125(1)
3	500	538	80	65	550	208(2)	95(1)	101(2)	0.0830(6)	0.089(1)
4	750	780	65	51	242	190(3)	98(1)	107(2)	0.1131(7)	0.125(2)

Supplementary Table I | Summary of sample parameters. For each sample the table gives the nominal lithographic ring radius $R_{\rm nom}$ and width $w_{\rm nom}$, as well as the values $R_{\rm GL}$ and $w_{\rm GL}$ obtained as global fit parameters. The number of rings on each cantilever is N. ξ_0, $\lambda_{\rm P0}$, and $B_{\rm c3,0}$ are the zero temperature values of the coherence length, Pearl penetration depth, and critical field $B_{\rm c3}$ determined from the fits in Fig. 2 in the main Text. The penetration depth $\lambda_0 = \sqrt{\lambda_{\rm P0}\, d}$. $B_{\rm c3,0}^{\rm GL}$ is calculated using ξ_0 and $w_{\rm GL}$, as described in the text. The quoted error in the final digit of each fit value corresponds to the statistical uncertainty of the fit (one standard deviation).

Supplementary Note 1. Background removal

We measure the shift of the resonant frequency of the cantilever f as function of field B. An example of raw data taken for a ring with radius $R = 406$ nm at temperature $T = 762$ mK is shown in Supplementary Figure 1a. Red trace corresponds to the sweep up of bias field and blue to the sweep down. In addition to the sawtooth oscillations associated with the rings' superconductivity, we observe that f also undergoes a small drift as a function of time and of B. To remove this background, we fit the data above the rings' critical field to a third-order polynomial, which is shown as the black curve in Supplementary Figure 1a. We subtract this fit from f to obtain the frequency shift df due to the magnetic moment of the rings (Supplementary Figure 1b).

Supplementary Note 2. Supercurrent as function of field

In our measurement configuration the magnetic field is perpendicular to the rings' surface and the frequency shift is related to supercurrent I as $df(B) = \kappa\, I(B)\, BR^2\pi$, where κ is a cantilever-specific constant [1, 2]. This constant depends on the cantilever's resonant frequency, spring constant, length and the number of rings on it. The resonant frequency is measured in the phase-locked loop, the length is measured by optical imaging, and the number of rings is known from the lithography pattern. The spring constant k is obtained as a fitting parameter of the Ginzburg-Landau fit, as explained in the main text and here in the following section. The best-fit value is within 20% of the nominal value computed as $k = (2\pi f)^2 m_{\text{eff}}$, where $m_{\text{eff}} = m/4$ is the effective mass of the cantilever and m is the cantilever's actual mass. The rings' radius is also obtained from the Ginzburg-Landau fits. It is highly constrained by the period of Aharonov-Bohm oscillations, with the result that the statistical error on the best-fit value is ≈ 1 nm. The values returned by this fit agree well with the values measured by SEM observations.

The supercurrent obtained from the frequency shift as $I(B) = df(B)/(2\pi\kappa R^2 B)$ is shown in Supplementary Figure 2. Every panel shows data measured on a sample with a different ring size in the full available temperature range.

The signal becomes very noisy close to zero field, and is not displayed for B very close to 0. This is because $I \propto df/B$ and for fields close to zero, dividing the signal df by B leads to unreliable results.

Supplementary Note 3. Little-Parks regime

As mentioned in the main text, at high T and high B the rings exhibit the Little-Parks effect: as function of bias flux the rings alternate between the superconducting and the normal state due to the competition between the superconducting condensation energy and the kinetic energy of the current imposed by bias flux [3, 4]. This can be seen directly in the $I(B)$ curves close to the rings' critical field, as they show regions of zero current (normal state) between regions of non-zero current (superconducting state). This is illustrated in Supplementary Figure 3, where the normal state regions are denoted by black arrows.

It is known from the Little-Parks effect that the supercurrent velocity, and therefore also supercurrent, reaches zero when bias flux $\Phi_{\text{min}} = n\Phi_0$, where n is an integer [3–5]. GL theory shows this is the case not only in the Little-Parks region, but in the full field region. Due to finite ring width there is a small correction on this condition [6] and in fact supercurrent is zero when

$$\Phi_{\text{min}} = \frac{n\Phi_0}{1 + \left(\frac{w}{2R}\right)^2}. \tag{1}$$

The correction $(w/2R)^2$ is very small in our experiment (a few percent), but at high winding number n it may lead to an observable deviation from the integer value. This is shown in Supplementary Figure 4 where full squares are the measured flux values at which the current goes to zero and the full black curve in Supplementary Figure 4a is the theoretical prediction (Supplementary Equation (1)), using parameters w and R obtained by the GL fit. In Supplementary Figure 4b a linear background $n\Phi_0$ has been subtracted from the data and from the theoretical prediction, and one directly sees the small linear slope due to the finite-width correction. At this scale we can distinguish red (sweep up) from blue (sweep down) data. The slight discrepancy between data and theory probably reflects the imperfect background subtraction in the $df(B)$ data, causing the observed zero current to deviate slightly from the actual value. Ideally, measured sweep up and sweep down values should coincide, so the scatter between them also points to noise in the readout.

Also shown in Supplementary Figure 4 with empty squares are measured flux values at which the winding number changes by one in the Little-Parks region. Those flux values are expected to be very close to $(n + 1/2)\Phi_0$. Again

there is a small correction of the order $(w/2R)^2$ which is too cumbersome to write explicitly. The black dotted line is the theoretical prediction including this correction. Supplementary Figure 4b shows this same data and theory as Supplementary Figure 4a, with the linear contribution $\Phi = n\Phi_0$ subtracted. Vertical purple lines show the extent of the normal regions (denoted by black arrows in Supplementary Figure 3).

Supplementary Note 4. Ginzburg-Landau fit for a one dimensional ring with finite width

We fit the data using a theory which includes the effects of finite ring width. More specifically, we use the expression for supercurrent $I(B)$ as given in Eq. (7) of [6]. That expression uses the relation of $I(B)$ to the coherence length ξ and field penetration depth λ_P prescribed by the Ginzburg-Landau theory, but does not specify the temperature dependence of ξ and λ_P. The latter dependence is found from the fits to the frequency shift data *df* taken in a broad range of temperatures and fields ($I(B)$ and *df* are related to each other via the spring constant). For brevity, we will refer to that procedure as to the Ginzburg-Landau fit.

To perform the fit, we first identify the winding number of each segment of $df(B)$. For the measurements taken with increasing B, we count the number of segments (i.e., the regions of smoothly varying *df* between jumps) between B_{c3} and $-B_{c3}$. This number is $2n_{\max} + 1$, where $n_{\max}$ is the maximum winding number. We thus determine $n_{\max}$. Then we start from B_{c3} and count down from $n_{\max}$ to zero. We apply the equivalent process to measurements taken with decreasing B.

As explained in the main text, it is a global fit which fits the entire $I(B)$ measurement (i.e., for all winding numbers and for B increasing and decreasing). The fitting parameters are: superconducting coherence length ξ, penetration depth λ, ring radius R, ring width w and spring constant k. Of these, we expect R, w and k to be fixed for each sample (i.e., to not change with temperature), so we first undertake a preliminary fit to determine these three parameters. In these preliminary fits, there is a degeneracy between λ and k, since they both set the amplitude of the signal: λ affects the condensation energy, and therefore the amplitude of the current, while k affects the proportionality constant κ between current and frequency shift. Therefore we first set the starting value $k_{\rm in}$ to its calculated nominal value (using the expression given in the Supplementary Note 2) and $\lambda_{\rm in}$ such that B_{c0} (the zero temperature bulk critical field, set by the product of ξ_0 and λ_0, where zeroes denote the zero temperature value) is 0.01 T, as expected for aluminium [5]. Then we run the fit for each of the $I(B)$ measurements (i.e., at different T) for that sample. We then fix k to be the mean of the values returned by these preliminary fits. Values for R and w are fixed in the same way: by picking the mean of the values obtained from fits at different temperatures. The scatter between the obtained values for k, R and w at different temperatures is rather small (a few percent for k and w and less than 1 nm for R).

In the second round of the fit only two fitting parameters remain, ξ and λ_P. Note that ξ also affects the condensation energy, and therefore the amplitude of the signal, but it is not degenerate with k and λ_P since it is very accurately set by the rings' critical field $B_{c3} \propto \xi^{-1}$, as detailed in the main text. This is in a sense lucky because our subsequent conclusions on the switching flux value hinge on the precise determination of ξ. This can be seen from Eqs. (1) and (2) in the main text which show that the switching flux criteria depend only on R and ξ. The temperature dependence $\xi(T)$ and $\lambda_P(T)$ found this way agrees well with the expectations based on theory and on earlier measurements of the thin-film critical field, see the main text.

We have made measurements for $T > 400$ mK. We have tried fitting below 750 mK ($\sim T_c/2$) but we have found that the values of w, λ_0 and ξ_0 don't converge to a fixed value like they do for $T > 750$ mK. This likely reflects the decreasing applicability of GL theory at lower temperatures.

The result of the measurement and the Ginzburg-Landau fit are shown in Supplementary Figures 5 and 6, where data is shown as points connected by thin curves and the fit is shown as thick curves. Three temperatures spanning the whole measured range are shown for each ring size. Red curves on each panel are for sweep up and blue for sweep down. The dotted black curves show the fit results extended over the full field range for each winding number; note that the portion of the dotted black curve occupied when B is increasing (red) is different from the part occupied for when B is decreasing (blue) in the hysteretic part of $I(B)$.

The most pronounced discrepancy between the data and the fit is found for the biggest ring at the lowest temperatures, see Supplementary Figure 5a, where the ring's self-inductance L starts to play a role. In this regime, we estimate $LI \sim 0.13\,\Phi_0$, which may lead to non-negligible skewing of the rings' current-phase relation [7]. For the rest of the measurements considered here, the effects of L are unimportant, i.e. $LI \ll \Phi_0$. For the smallest to largest ring size we have computed the expected $L = 0.5 - 2.3$ pH. This gives $LI \sim 0.03, 0.05, 0.07, 0.13\,\Phi_0$ respectively at 750 mK. At higher temperatures LI is less since I decreases with temperature.

Supplementary Note 5. Sample parameters

Sample parameters are listed in Supplementary Table I.

The values of ξ_0 and $B_{c3,0}$ from Figure 2a and c in the main text and given in the Supplementary Table I can be compared against two separate estimates. First, we note that ξ_0 can also be determined via transport measurements, using the relationship $\xi_0 = 0.855\sqrt{\xi_0^b l_e}$ [5], where $\xi_0^b = 1.6\,\mu$m is the bulk Al coherence length and l_e the electron mean free path. Transport measurements of Al wires that were co-deposited with the rings studied here give $l_e = 35 \pm 5$ nm [1]; this corresponds to $\xi_0 = 205 \pm 15$ nm, in close agreement with the values inferred from the measurements of $I(B)$. Second, we note that $B_{c3,0}$ can be calculated directly from $B_{c3,0} = 3.67\Phi_0/(2\pi w \xi_0)$ using the values of ξ_0 determined from the fits in Figure 2a in the main text and $w_{\rm GL}$. The results of this approach are listed in Supplementary Table I as $B_{c3,0}^{\rm GL}$. For each sample, $B_{c3,0}^{\rm GL}$ and $B_{c3,0}$ agree to $\approx 10\%$.

Figure 2 in the main text shows that ξ and λ_P obtained as fitting parameters follow the expected empirical temperature dependence, while measured rings' critical field B_{c3} follows the Ginzburg-Landau prediction. The critical temperature is obtained as a fitting parameter from all three panels separately, and it is found to be: $1.316 \pm 0.001, 1.391 \pm 0.004$ and 1.318 ± 0.002 K respectively. We see that the critical temperature obtained from the fit of λ_P is larger by 5 %. This is possibly due to the fact that at low temperature the thickness of the rings is not much larger than λ and the system is marginally in the regime where Pearl penetration depth applies.

Supplementary Note 6. Free energy

With values of $\xi(T)$ and $\lambda_{\rm P}(T)$ obtained by fitting $I(B)$, it is straightforward to calculate the free energy $F_n(B)$ of each equilibrium state [6]. The black line in Supplementary Figure 7a shows $F_n(B)$ for the rings with $R = 406$ nm and $T = 876$ mK. In Supplementary Figure 7a, the red (blue) curves show the path taken by the rings as B is increased (decreased). The path is determined by using the values of n inferred from the data. Supercurrent is related to the free energy as $I \propto -\partial F/\partial B$.

Supplementary Figure 7a shows that the phase slips for increasing and decreasing B are located nearly symmetrically around the minima of $F_n(B)$. Closer inspection shows that the phase slips occur near the inflection points of $F_n(B)$. To examine the location of these phase slips quantitatively, in the main text we define $\Delta\phi_n^{\pm} = \phi_n^{\pm} - \phi_{\min,n}$, where $\phi_n^{\pm}$ is the experimental value of the normalized flux $\phi = \Phi/\Phi_0$ at which the transition $n \rightleftarrows n \pm 1$ occurs, and $\phi_{\min,n}$ is the value of ϕ at which F_n reaches its minimum value (or, as stated in the main text, where the current $I_n \propto -\partial F_n/\partial\phi$ reaches zero, see also Supplementary Eq. (1)). As defined, $\Delta\phi_n^{+}$ are positive (increasing B) and $\Delta\phi_n^{-}$ are negative (decreasing B).

Both in a current-biased wire and in a flux-biased ring the phase of the order parameter at equilibrium is $\phi = ks$, where k is a wave-vector, and s the longitudinal coordinate along the wire or ring. Supercurrent is then $I \propto k(1-k^2)$ [6, 8]. The boundary condition for the wire is $kL = 2\pi n$, where L is the wire length, and for a ring $kL + 2\pi\phi = 2\pi n$, where $L = 2\pi R$. When biasing a wire with current $I < I_c$, k is not uniquely determined since $I \propto k(1-k^2)$ has multiple solutions, and the system will always chose the value of k in the stable region (non-shaded area in Supplementary Figure 7b-d). (Here "stable" refers to the long wire/ring diameter limit). In contrast, when biasing a ring with flux, k is uniquely determined (through the boundary condition), and therefore it is possible to bias the system in the shaded region, which corresponds to the regions indicated by black arrows in Figure 4 in the main text. In these regions the velocity is super-critical (see Supplementary Figure 7d), but the diminishing density leads to the decrease of current. This effect is only accessible in the the ring configuration.

Supplementary Note 7. Phase slip flux

In Supplementary Figure 8 we show the extended version of Figure 3 from the main text. Data for each ring size is given in a separate panel. The measured transition widths are denoted by bars. Dotted lines are the theoretically predicted values of the switching flux in the limit of a long sample (Eq. (1) in the main text) and full lines are the theory prediction which takes into account the finite-length correction (Eq. (2) in the main text).

The largest disagreement between data and prediction occurs for the largest rings at low temperature ($R = 780$ nm, blue and violet squares in Supplementary Figure 8d). This discrepancy is likely due to the increased importance of the rings' self-inductance in this regime, which is ignored in our analysis. Self-inductance leads to the skewing of the current-phase relation and as consequence the GL fit doesn't work as well.

Supplementary Note 8. Transition width

We have also studied the width of the jumps from one winding number to another, which is non-zero since the measurement is performed on an array of rings. The result is shown in Supplementary Figure 9 where the transition widths are given as function of the winding number n, proportional to B, for all ring sizes and temperatures. We see that the transition widths have a non-zero value at $n = 0$ and increase roughly linearly with B. The slope of the B-dependence is independent of T and decreases with R. It is consistent with lithographic ring-to-ring imprecision $\Delta R = 1.9$, 1.5, 2.1 and 2.0 nm for rings with $R = 780$, 538, 406 and 288 nm respectively.

The transition width at zero field also shows no discernible temperature dependence. It is a factor of $5-10$ larger than expected from the rings' mutual inductance, which results in rings at the middle of an array seeing a slightly different field than those at the edge. Extrapolating the linear behavior of the transition width to zero field gives $\Phi_{n=0}$, which is seen to increase with R.

We conclude from these observations that the rings' temperature does not influence the transition widths of the arrays. This is consistent with the fact that the transition width expected for thermal switching across a barrier [9] is estimated to be several times less than the observed width.

Supplementary Note 9. Estimate of damping at low temperatures

As explained in the main text, the presence of a large supercurrent justifies the use of the time-dependent Ginzburg-Landau theory at $T \to T_\mathrm{c}$. In that approximation, the time evolution of the phase difference across the phase slip is described by viscous "motion" of the phase. As a result, $\Delta n = 1$ in a deterministic phase slip at T close to T_c. Since there is no quantitative theory of deterministic phase slips at low temperature, we proceed with estimates helping to asses the possibility of $\Delta n > 1$.

At high winding numbers, the dependence of the kinetic energy of the moving condensate on current I can be approximated by the linear function $\delta E = (\hbar/e)I$. The deterministic phase slip occurs once I reaches its critical value, $I = I_\mathrm{c}$. Therefore, in a phase slip the condensate energy changes by $E_{\Delta n} = (\Delta n\hbar/e)I_\mathrm{c}$. The critical current $I_\mathrm{c} = Sj_\mathrm{c}$ is proportional to the cross-sectional area S of the wire making the ring. The critical current density j_c can be estimated from the Usadel [10] and BCS self-consistency equations [5] that allow one to relate j_c to the gap width Δ, the electron diffusion coefficient, and the electron density of states at the Fermi level. As a result, at low temperatures $j_\mathrm{c} \sim \Delta/(e\rho\,\xi)$ is expressed in terms of the corresponding values of Δ and coherence length ξ, and the normal-state resistivity ρ (we note that j_c acquires an additional factor $(1-T/T_\mathrm{c})^{3/2}$ at $T \to T_\mathrm{c}$, in accordance with the GL theory). Thus, at low temperatures we find for the condensate energy difference

$$E_{\Delta n=1} \sim \frac{\hbar}{e^2}\frac{S}{\rho\,\xi}\Delta\,. \tag{2}$$

The second factor here, $G = S/(\rho\,\xi)$, has the meaning of the normal-state conductance of a wire segment long enough to house a phase slip.

The barrier "protecting" the lower metastable state is $\delta F = (\hbar/e)I_\mathrm{c}\sqrt{3/2}\,(1-j/j_\mathrm{c})^{5/4} = E_{\Delta n=1}\sqrt{3/2}\,(1-j/j_\mathrm{c})^{5/4}$ [8]. As already mentioned in Supplementary Note 6, $I \sim j \sim k(1-k^2)$, where k is the wave-vector of the superconducting phase (here in the units of $1/\xi$). Close to I_c ($k \to 1/\sqrt{3}$), we have $1 - j/j_\mathrm{c} \sim (9/2)\left(k - 1/\sqrt{3}\right)^2$, which, in combination with $k = (\xi/R)(n-\phi)$, yields $\delta F_{\Delta n=1} \sim (\xi/R)^{5/2}\,E_{\Delta n=1}$.

To estimate the dissipation due to the quasiparticle production in the course of a phase slip, we model it as a short SNS junction of conductance G. A finite phase difference φ across it results in the appearance of Andreev levels with sub-gap energies. Furthermore, the time dependence of φ leads to Landau-Zener transitions between the occupied and empty levels. As the result, an out-of-equilibrium level occupation is created. In a $\Delta n = 1$ phase slip, the phase difference φ starts from 0 and ends at 2π; respectively, the Andreev levels "peel off" and merge with the edges of the gap Δ. At the end of the cycle, the non-equilibrium occupation of levels transforms into pairs of free quasiparticles each pair carrying energy 2Δ. The number of created pairs N_p depends on $d\varphi/d\tau$ (τ denotes time) in the course of the phase slip. To estimate it, we use the result [11] developed for the dissipative current across a short SNS junction, $I_\mathrm{diss} = G\sqrt{|V|\Delta/e}$ at a constant low ($eV \ll \Delta$) bias, $V = (\hbar/2e)(d\varphi/d\tau)$. For estimates, we set $|d\varphi/d\tau| \sim 2\pi/\tau_\mathrm{ps}$ with τ_ps being the time it takes to undergo a phase slip. Dispensing with the unreliable numerical factors, the energy spent on the quasiparticles' production can be estimated as

$$E_\mathrm{diss} = 2\Delta N_p \sim \Delta\tau_\mathrm{ps}I_\mathrm{diss}/e \sim \frac{\hbar}{e^2}G\Delta\left(\Delta\tau_\mathrm{ps}/\hbar\right)^{1/2}\,. \tag{3}$$

Lastly, we use the estimate $\tau_{\rm ps} \sim \hbar/\Delta$. It may be viewed as the extrapolation of the TDGL characteristic time $\sim \hbar/|T - T_{\rm c}|$ to low temperatures, or as the RC time constant of the junction with the capacitance $C \sim \hbar G/\Delta$ renormalized by the quantum fluctuations of charge [12]; the two approaches yield the same result. Replacing $\tau_{\rm ps} \to \hbar/\Delta$ and using the conductance $G = S/(\rho\,\xi)$ associated with the phase slip in Supplementary Equation (3), we find $E_{\rm diss} \sim (\hbar S/e^2\rho\,\xi)\Delta$ quoted in the main text.

Supplementary References

[1] Bleszynski-Jayich, A. C. *et al.* Persistent currents in normal metal rings. *Science* **326**, 272-275 (2009).
[2] Shanks, W. E. Persistent currents in normal metal rings. *Thesis* (Yale University, 2011).
[3] Little, W. A. & Parks, R. D. Observation of quantum periodicity in the transition temperature of a superconducting cylinder. *Phys. Rev. Lett.* **9**, 9-12 (1962).
[4] Parks, R. D. & Little, W. A. Fluxoid quantization in a multiply-connected superconductor. *Phys. Rev.* **133**, A97-A103 (1964).
[5] Tinkham, M. *Introduction to Superconductivity* 2nd edn (Dover, 2004).
[6] Zhang X. & Price, J. C. Susceptibility of a mesoscopic superconducting ring. *Phys. Rev. B* **55**, 3128-3140 (1997).
[7] Fink, H. J. & Grünfeld, V. Flux periodicity in superconducting rings: Comparison to loops with Josephson junctions. *Phys. Rev. B* **33**, 6088-6093 (1986).
[8] Langer, J. S. & Ambegaokar, V. Intrinsic resistive transition in narrow superconducting channels. *Phys. Rev.* **164**, 498-510 (1967).
[9] McCumber, D. E. & Halperin, B. I. Time scale of intrinsic resistive fluctuations in thin superconducting wires. *Phys. Rev. B* **1**, 1054-1070 (1970).
[10] Usadel, K. D. Generalized Diffusion Equation for Superconducting Alloys. *Phys. Rev. Lett.* **25**, 507-509 (1970).
[11] Bardas, A. & Averin, D. V. Electron transport in mesoscopic disordered superconductor – normal - metal – superconductor junctions. *Phys. Rev. B* **56**, R8518-R8521 (1997).
[12] Larkin, A. I. & Ovchinnikov, Yu. N. Decay of the supercurrent in tunnel junctions. *Phys. Rev. B* **28**, 6281-6285 (1983).
